# Genomic identification and expression analysis of acid invertase (AINV) gene family in *Dendrobium officinale* Kimura et Migo

**DOI:** 10.1186/s12870-024-05102-8

**Published:** 2024-05-14

**Authors:** Yujia Liu, Boting Liu, Kefa Luo, Baiyin Yu, Xiang Li, Jian Zeng, Jie Chen, Rui Xia, Jing Xu, Yuanlong Liu

**Affiliations:** 1 Guangdong Province Key Laboratory of Utilization and Conservation of Food and Medicinal Resources in Northerrn Region, Shaoguan University, Shaoguan, Guangdong 512005 China; 2 State Key Laboratory for Conservation and Utilization of Subtropical Agro-Bioresources, South China Agricultural University, Guangzhou, 510642 China; 3 College of Horticulture, South China Agricultural University, Guangzhou, 510642 China; 4 College of Biology and Agriculture, Shaoguan University, Shaoguan, Guangdong 512005 China

**Keywords:** *Dendrobium officinale*, Acid invertase, Genomic identification, Expression analysis, Abiotic stress, Protein–protein interaction

## Abstract

**Background:**

*Dendrobium officinale* Kimura et Migo, a renowned traditional Chinese orchid herb esteemed for its significant horticultural and medicinal value, thrives in adverse habitats and contends with various abiotic or biotic stresses. Acid invertases (AINV) are widely considered enzymes involved in regulating sucrose metabolism and have been revealed to participate in plant responses to environmental stress. Although members of AINV gene family have been identified and characterized in multiple plant genomes, detailed information regarding this gene family and its expression patterns remains unknown in *D. officinale*, despite their significance in polysaccharide biosynthesis.

**Results:**

This study systematically analyzed the *D. officinale* genome and identified four *DoAINV* genes, which were classified into two subfamilies based on subcellular prediction and phylogenetic analysis. Comparison of gene structures and conserved motifs in *DoAINV* genes indicated a high-level conservation during their evolution history. The conserved amino acids and domains of DoAINV proteins were identified as pivotal for their functional roles. Additionally, cis-elements associated with responses to abiotic and biotic stress were found to be the most prevalent motif in all *DoAINV* genes, indicating their responsiveness to stress. Furthermore, bioinformatics analysis of transcriptome data, validated by quantitative real-time reverse transcription PCR (qRT-PCR), revealed distinct organ-specific expression patterns of *DoAINV* genes across various tissues and in response to abiotic stress. Examination of soluble sugar content and interaction networks provided insights into stress release and sucrose metabolism.

**Conclusions:**

*DoAINV* genes are implicated in various activities including growth and development, stress response, and polysaccharide biosynthesis. These findings provide valuable insights into the AINV gene amily of *D. officinale* and will aid in further elucidating the functions of DoAINV genes.

**Supplementary Information:**

The online version contains supplementary material available at 10.1186/s12870-024-05102-8.

## Background

*Dendrobium officinale* Kimura et Migo, known as *Dendrobium catenatum*, is an endangered perennial herbal plant belonging to the Orchidaceae family, endemic to China, and esteemed for its significant ornamental and medicinal values [1, 2]. As an epiphytic plant, wild *D. officinale* usually grows in challenging environments, such as perching on cliffs or tree trunks, and distributed at altitudes exceeding 1,200 m [3]. While it naturally thrives in, warm and humid climates, *D. officinale* faces inevitable abiotic stresses, including low temperature and drought, leading to reduced growth and yield [4, 5]. However, even in such harsh habitats, *D. officinale* exhibit well growth and accumulate important medicinal compounds due to their evolved stress tolerance system. Moreover, *D. officinale* ranks the top position among nine Chinese herbs for longevity and has had a documented use in folk medicine for over 1300 years [6]. Over recent decades, various bioactive constituents, including polysaccharides, bibenzyls, alkaloids, and flavones, have been identified and isolated from *D. officinale* plants [7, 8]. Notably, polysaccharides in stems of *D. officinale* serve as crucial active ingredient, exhibiting antioxidant [9], antitumor [1], and anti-inflammatory properties [10], while also supporting immune modulation [11], hepatic protection [12], and hypoglycemic effects [13], resulting in their high commercial values as traditional medicines [14, 15].

Sucrose, the primary product of photosynthesis and the principal form of carbohydrate transport, is an essential element of the life cycle in higher plants [16]. In *D. officinale*, polysaccharides are synthesized through the hydrolysis of sucrose, which necessitates the involvement of multiple protein or enzymatic genes [17]. The sucrose hydrolysis depends on two critical gene families: sucrose synthase (SUS, EC 2.4.1.13) and invertase (INV, EC 3.2.1.26) [18]. While SUS catalyzes the reversible conversion of sucrose and uridine diphosphate (UDP) into uridine diphosphate glucose (UDP-glucose) and fructose, INV irreversibly hydrolyze sucrose into glucose and fructose. Despite SUS’s crucial role in mumerous metabolic pathways, INV surpasses SUS in its significance for sucrose hydrolysis. Previous studies into *D. officinale* polysaccharides primarily focused on their chemical composition and pharmacological activities. However, polysaccharide biosynthesis at the molecular level in *D. officinale* remains unclear, especially concerning gene function and transcriptional regulation. As an essential enzyme for polysaccharides synthesis, INV promotes the accumulation of polysaccharides through sucrose hydrolysis, thereby modulating various biological processes including growth and development [19, 20], stress resistance [21], signal transduction, and the biosynthesis of secondary metabolites [22, 23].

A multigene family in plants encodes INV proteins. According to their optimal pH value, solubility, and subcellular localization, INVs are primarily classified into three types: cytoplasmic invertase (CINV), insoluble cell wall invertase (CWINV), and soluble vacuolar invertase (VINV) [24]. CINV belongs to a neutral/alkaline invertase (NINV), whereas CWINV and VINV pertain to acid invertases (AINV), also referred to as β-fructofuranosidase, owing to their ability to hydrolyze sucrose and other β-fructose oligosaccharides [25, 26]. Moreover, AINV can hydrolyze fructose-containing compounds apart from sucrose, such as raffinose and stachyose, which are strongly inhibited by heavy metals [24]. The initial three AINV proteins isolated from carrot (*Daucus carota*) were encoded by distinct genes [27]. In recent years, as more plant genomes have been assembled and reported, AINV gene families have been identified across numerous plant species, including *Arabidopsis thaliana* [28], rice (*Oryza sativa*) [29], sweet sorghum (*Sorghum Bicolor*) [30], and poplar (*Populus trichocarpa*) [31].

Prior reports have suggested that the number of genes encoding AINV proteins varies among different species, and they are expressed independently at specific stages and tissues during growth and development [32, 33]. For instance, two VINV genes, named *Atβfruct3* and *Atβfruct4*, displayed distinct expression patterns during organ development across various organs of *A. thaliana* [28]. Similarly, the two VINV genes exhibited differential expression in carrot, with *SI* primarily expressed in primary roots and *SII* exclusively expressed, playing a central role in root tip development [27]. *GhVIN1* demonstrated specific expression in the cotton (*Gossypium hirsutum*) seed coat, critical for pollination success and the fertility of paired male and female organs [33]. In other plant species such as tea (*Camellia sinensis*) [34], tomato (*Solanum lycopersicum*) [35], pepper (*Capsicum annuum*) [36], cassava (*Manihot esculenta*) [37], and potato (*Solanum tuberosum*) [38], AINV genes also exhibit tissue-specific expression patterns, implying their involvement in specialized physiological functions. Despite extensive exploration of, AINV genes in various species, little is known about these genes in *D. officinale*.

In addition, AINVs have also been identified to be involved in responses to environmental stress [28, 34]. For example, *StVIN1* was induced by low temperatures in mature potato tubers and played a crucial role in saccharification [38]. In poplar, the three AINV genes, *PtVINV1/2* and *PtVINV3*, were up-regulated under low-temperature treatment, while *PtVINV1/2* was also up-regulated under NaCl treatment [31]. Conversely, under drought stress, the expression of *AINV* genes in maize (*Zea mays*) ovaries was decreased. However, expression could be restored upon sucrose supplementation, suggesting the significant role of AINV in response to drought stress. Although extensive research on AINVs in various plant species, a comprehensive investigation of the AINV gene family in *D. officinale* (*DoAINV*) has not yet been undertaken. Therefore, the present study utilized bioinformatics methods to analyze the characteristics of *DoAINV* genes on a genome-wide scale in *D. officinale* based on publicly available data [6, 39]. Furthermore, we examined the expression profiles of the *DoAINV* genes in different tissues and their response to various abiotic stresses. These comprehensive findings will contribute to a better understanding of the potential functions of AINV enzymes in polysaccharide biosynthesis in *Dendrobium* plants for further research.

## Results

### Identification of the AINV gene family in *D. officinale*

To identify *DoAINV* genes, a Hidden Markov Model (HMM) was constructed by querying sequences against glycosyl hydrolases family domains (PF00251 and PF08244), which were then searched against the *D. officinale* genome. *A. thaliana* and rice AINV sequences served as queries in the BLAST program. Furthermore, The resulting sequences were confirmed using an online CDD-search tool, resulting in the identification of four putative AINV members in the *D. officinale* genome after removing redundant sequences. Following manual reannotation and confirmation of protein characteristic domain, the four *DoAINV* genes were designated *DoVIN1* and *DoVIN2*, belonging to the VINV sub-family, and *DoCWIN1* and *DoCWIN2*, belonging to the CWINV sub-family, consistent with the nomenclature proposed in previous study [29]. The basic characteristics of DoAINV members are presented in Table 1. The size of *DoAINV* genes varied from 3,345 bp to 13,803 bp, with coding DNA sequences (CDS) ranging from 1,725 bp to 1,983 bp. Molecular analysis of the full-length deduced polypeptides revealed that the putative proteins of DoAINVs ranged from 574 to 660 amino acid residues, with relative molecular weights and isoelectric points varying between 65.86 to 72.74 kDa, and 5.54 to 9.21, respectively. This range of variability implies that different *D. officinale* AINV proteins may operate in different microenvironments.

**Table 1:** Characteristics of *DoAINV* gene family members

Protein Type	Protein Symbol	Gene Name	Gene Symbol	Genomic DNA Size (bp)	CDS Size (bp)	Number of Amino Acids	Predicted Mw (kDa)	Theoretical pI	GRAVY	II	TMH	Localization prediction	Functional Domains (Start–End)	
													**a (N)**	**b (C)**
Vacuolar Invertase	XP_020703196.1	DoVIN1	LOC110114607	5527	1887	628	69.84	6.09	-0.184	38.33	1	Vacuole	97–421	424–617
	XP_020677523.2	DoVIN2	LOC110096097	5604	1983	660	72.74	5.54	-0.165	35.55	1	Vacuole	123–448	451–643
Cell Wall Invertase	XP_020686802.1	DoCWIN1	LOC110102702	3345	1725	574	66.40	5.85	-0.339	41.59	0	Cell wall	50–369	372–567
	XP_020678379.2	DoCWIN2	LOC110096666	13803	1758	585	65.86	9.21	-0.507	35.81	0	Cell wall	59–377	380–576

*CDS* Coding DNA sequence, *MW* molecular weight, *pI* isoelectric point, *GRAVY* grand average of hydropathicity, *II* Instability index, *TMH* transmembrane helix

a (N), glycosyl hydrolases family 32 N-terminal domain

b (C), glycosyl hydrolases family 32 C-terminal domain

**Fig. 1:**
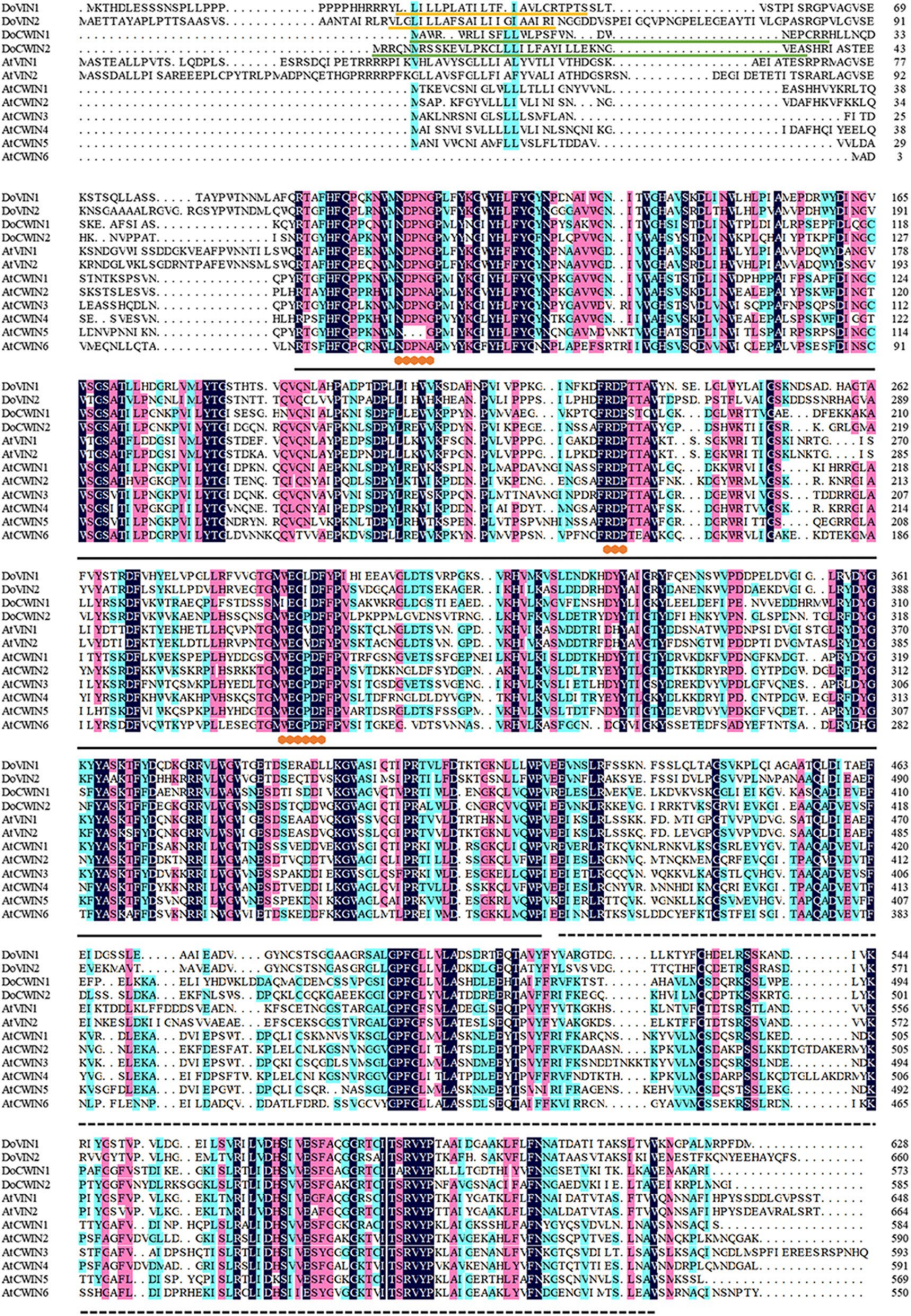
Sequence alignment of *AINV* genes from *A. thaliana* and *D. officinale*. Dark blue, pink, and light blue shading denote 100%, 75%, and 50% conservation of amino acid residue, respectively. Yellow lines indicate the predicted transmembrane domains. Green lines highlight the predicted signal peptides. Orange dots represent conserved motifs of NDPNG (β-fructosidase), FRDP, and WECXDF. The solid black line indicates the glycosyl hydrolases family GH32 N-terminal domain, and the black dashed line represents glycosyl hydrolases family GH32 C-terminal domain

Sequence comparison among DoAINVs revealed high sequence homology at both the nucleotide level (47.09% to 60.93% identity) within the coding region and at the amino acid level (37.02% to 65.76% identity) (Table 2). Notably, gene pairs *DoVIN1* and *DoVIN2*, and *DoCWIN1* and *DoCWIN2*, exhibited a higher sequence identity at the nucleotide and amino acid levels, respectively, indicating the division of DoAINVs into two evolutionary sub-families. Furthermore, two conserved domains, glycosyl hydrolases family 32 N-terminal and 32 C-terminal domains, recognized as typical plant AINV domains, were detected in all DoAINVs and *A. thaliana* AINVs (Fig. 1). Both four AINVs from *D. officinale* and eight AINVs from *A. thaliana* contained the catalytic motifs NDPNG, RDP, and WECXDF. These findings suggest that the four *DoAINVs* encode different AINV isozymes.

**Table 2:** Analysis of homology between DoAINV nucleotide and amino acid sequences

		Identity of amino acid sequences (%)			
		DoVIN1	DoVIN2	DoCWIN1	DoCWIN2
Identity of nucleotide sequences (%)	DoVIN1	-	65.76	39.48	42.14
	DoVIN2	60.93	-	37.02	40.75
	DoCWIN1	50.84	48.59	-	52.03
	DoCWIN2	50.94	47.09	58.90	-

**Fig. 2:**
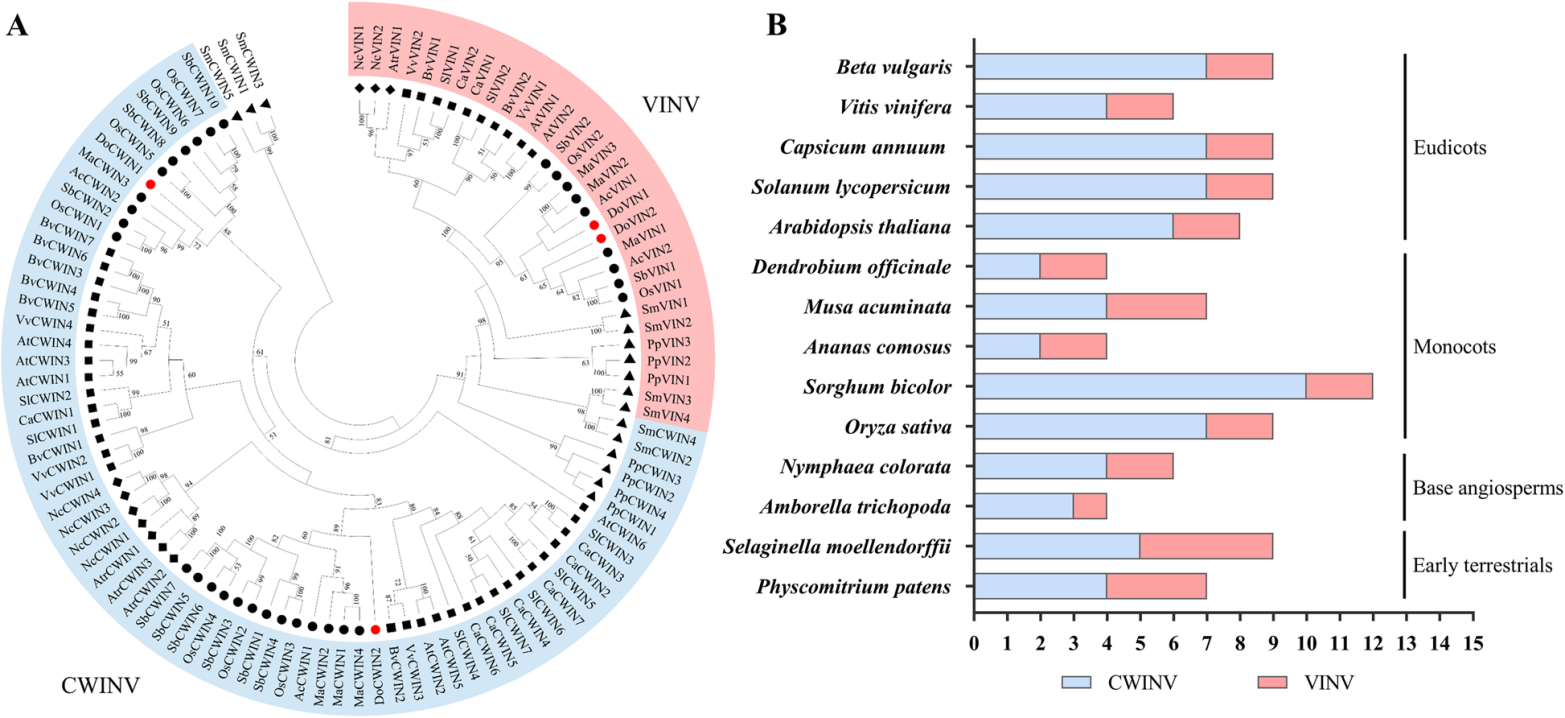
Phylogenetic analyses of AINVs from *D. officinale* and various plant species. **A** The phylogenetic tree was constructed using the neighbor-joining (NJ) algorithm with 1,000 bootstrap replicates. All AINV family genes were grouped into two subfamilies named VINV and CWINV. VINVs are colorized by the pink zone, CWINVs are colorized by the blue zone. Red points indicate *D. officinale* AINVs. Solid points indicate monocots, solid squares represent eudicots, solid rhombus indicate base angiosperms, and solid triangles represent early terrestrial plants. **B** AINV family members from different plant species: *B. vulgaris*, *V. vinifera*, *C. annuum*, *S. lycopersicum*, *A. thaliana*, *D. officinale*, *M. acuminata*, *A. comosus*, *S. bicolor*, *O. sativa*, *N. colorata*, *A. trichopoda*, *S. moellendorffii*,* P. patens*

To further elucidate the evolutionary relationship between AINV gene families from *D. officinale* and other species, 103 full-length amino acid sequences from 14 plant species (comprising 2 early terrestrial plants, 2 base angiosperms, 5 eudicots, and 5 monocots, Table S1) were aligned, and an unrooted phylogenetic tree was constructed (Fig. 2A). All 103 AINV proteins clustered into two major subfamilies according to the phylogenetic tree, designated as VINV and CWINV, consistent with the previous classification of the AINV family in rice and poplar [29, 31]. These groups could be further classified into early terrestrial, basal angiosperm, eudicotyledonous, and monocotyledonous sub-groups (Fig. 2B). The AINV gene family, with an ancient origin and no expansion through gene duplication beyond the event of polyploidization, has undergone both positive and negative selection pressures [31, 40]. These results suggest that the evolutionary divergence of the *AINV* genes could have occurred before the differentiation of dicot and monocot ancestor. DoVIN1 and DoVIN2 clustered in the VINV group, while DoCWIN1 and DoCWIN2 clustered in the CWINV group.

### Gene structure and conserved motifs of *D. officinale* AINVs

**Fig. 3:**
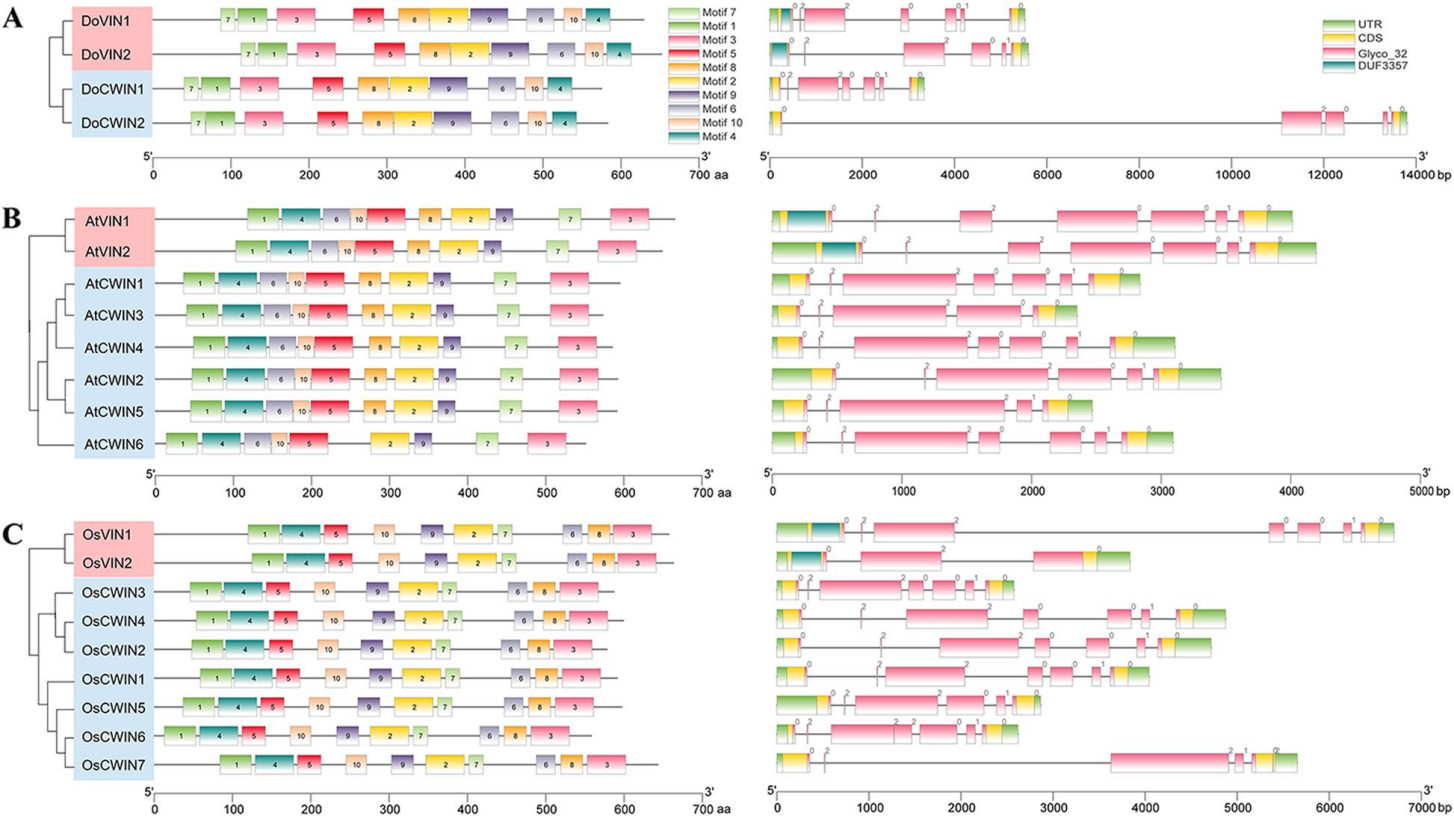
The conserved motifs and exon–intron organization analyses of plant *AINV* genes based on their evolutionary relationship. **A** Structural features of four *AINV* genes from *D. officinale*. **B** Structural features of eight *AINV* genes from the genome of *A. thaliana*. **C** Structural features of nine *AINV* genes from the rice (*Oryza sativa*) genome. Different colors on the left side denote conserved motifs, while different colors on the right side represent the exon–intron structures, and the numbers “0”, “1”, and “2” indicate the phases of introns. The phylogenetic tree between AINVs was constructed using MEGA 11

The distributions of protein-conserved motifs and gene intron-exons were comparatively analyzed across the genomes of *D. officinale*, *A. thaliana*, and rice to investigate the structural features and explore the evolutionary mechanisms of AINV gene families. Ten distinct motifs (motifs 1–10) were identified in the AINV protein sequences of these three plants, with the longest conserved motif spanning 50 amino acids (Table 3). While most motifs classified as AINVs exhibited similarity, their distribution patternss differed significantly among species. Notably, conserved motifs located within the N-terminal region in CWINVs were longer than those in VINVs (Fig. 3), suggesting common ancestral AINV genes between monocots and dicots at these positions.

**Table 3:**
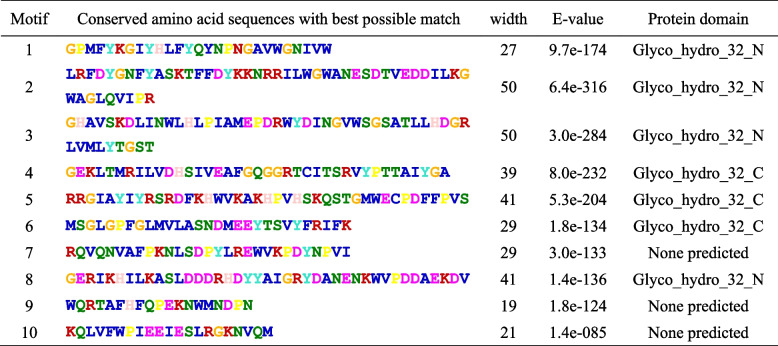
Putative conserved amino acid sequences of AINV motifs from *D. officinale* and A. *thaliana*

The exon–intron arrangement is considered a crucial parameter in gene phylogenies [41]. Analysis of *AINV* gene structures in *D. officinale*, *A. thaliana*, and rice revealed variation in the number of introns, ranging from four to six. Similarly, the number of exons ranged from five to seven, with the exception of a rice member (Os02g01590.1, Fig. 3C). Most *AINV* genes, except *DoCWINV2* and *Os02g01590.1*, included a mini exon consisting of nine nucleotides. The mini exon encoded aspartic acid, proline, and asparagine, ranking second, and was situated within the β-fructosidase motif (NDPNG). Additionally, all *AINV* genes contained a typical Glyco_32 domain belonging to the glycosyl hydrolases family 32. Only VINV genes possessed an additional domain of unknown function (DUF3357). Phylogenetic analysis of the four *DoAINVs* revealed their classification into two distinct groups (Fig. 3A), consistent with the pattern observed in the phylogenetic tree of AINV sequences (Fig. 2A). These findings support the specific evolutionary characteristics of the AINV gene family across different plant species.

### Prediction of the protein structure of *D. officinale* AINV proteins

The secondary structure analysis revealed that the four DoAINV proteins consisted of *α*-helices, extended *β* strands, *β*-turns, and random coils (Fig. S1, Table 4). Random coils were the predominant secondary structures among the DoAINV proteins, accounting for 51.62 to 55.41%, followed by extended *β* strands (23.76 to 26.88%). The *α*-helices were less prevalent, ranging from 13.38 to 18.97%, implying that the DoAINV proteins were highly unstable and susceptible to degradation (Table 4). These distributions of secondary structures were also highly conserved across four DoAINV polypeptide chains (Fig. S1).

**Table 4:** Secondary structural statistics of DoAINV proteins

Protein	Alpha helix (%)	Extended Beta strand (%)	Beta turn (%)	Random coil (%)
DoVIN1	13.38	25.8	5.41	55.41
DoVIN2	17.42	24.09	4.7	53.79
DoCWIN1	15.01	26.88	5.93	52.18
DoCWIN2	18.97	23.76	5.64	51.62

**Fig. 4:**
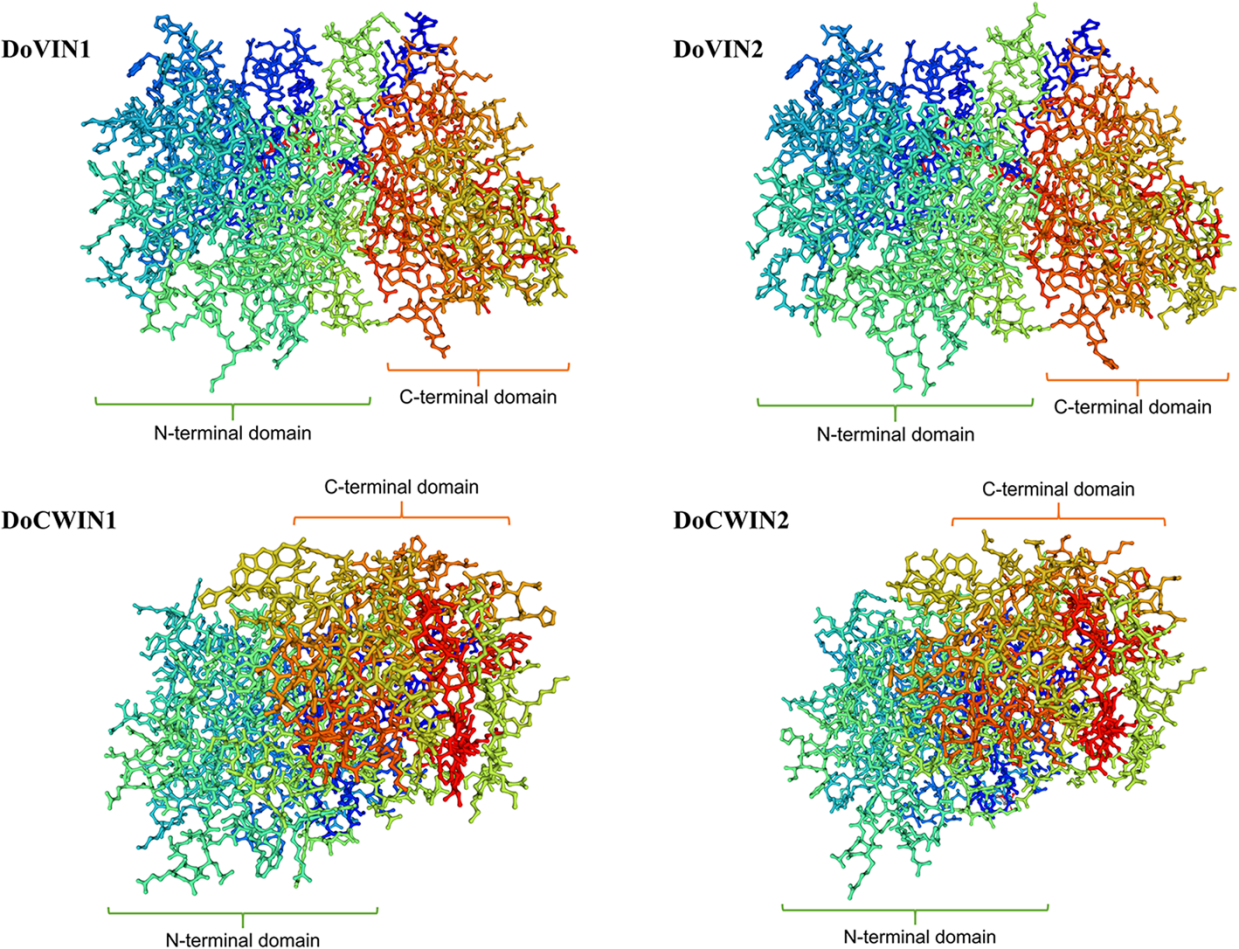
Predicted tertiary structure models of DoAINV proteins

Structures of the four DoAINV proteins were predicted using the Swiss-model online software (Fig. 4), and their tertiary dimensional models (3D) were based on templates 3ugf.1.A (6-fructosyltransferase) and 2ac1.1.A (invertase). Each DoAINV tertiary structure consisted of a single monomer, comprising a single main polypeptide chain. The 3D structures DoVIN1 and DoVIN2 exhibited similarity, while DoCWIN1 was closer to DoCWIN2.

Prediction of the transmembrane structure (Fig. S2) indicated that DoVIN1 and DoVIN2 protein sequences contained a high reliable hydrophobic region at the N-terminal; however, DoCWIN1 and DoCWIN2 did not (Fig. 1). Conversely, the SignalP-5.0 prediction results showed that DoCWIN1 and DoCWIN2 contained one signal peptide composed of 19 to 27 amino acids and 34 amino acids, respectively, with reliabilities of 0.5201 and 0.939, while no signal peptide was detected in DoVIN1 and DoVIN2 proteins (Fig. 1 and Fig. S3). This implies that DoVIN1 and DoVIN2 might function as membrane proteins in the endoplasmic reticulum, whereas DoCWIN1 and DoCWIN2 might act as secreted proteins. Furthermore, phosphorylation is crucial for protein activity, function, and intracellular signal transduction. Prediction of phosphorylation sites in DoAINV proteins indicated that serine was the most common site for phosphorylation in all DoAINVs, followed by threonine and tryptophan phosphorylation sites (Fig. S4).

### *Cis*-elements analysis in promoter regions of *D. officinale* AINV genes

**Fig. 5:**
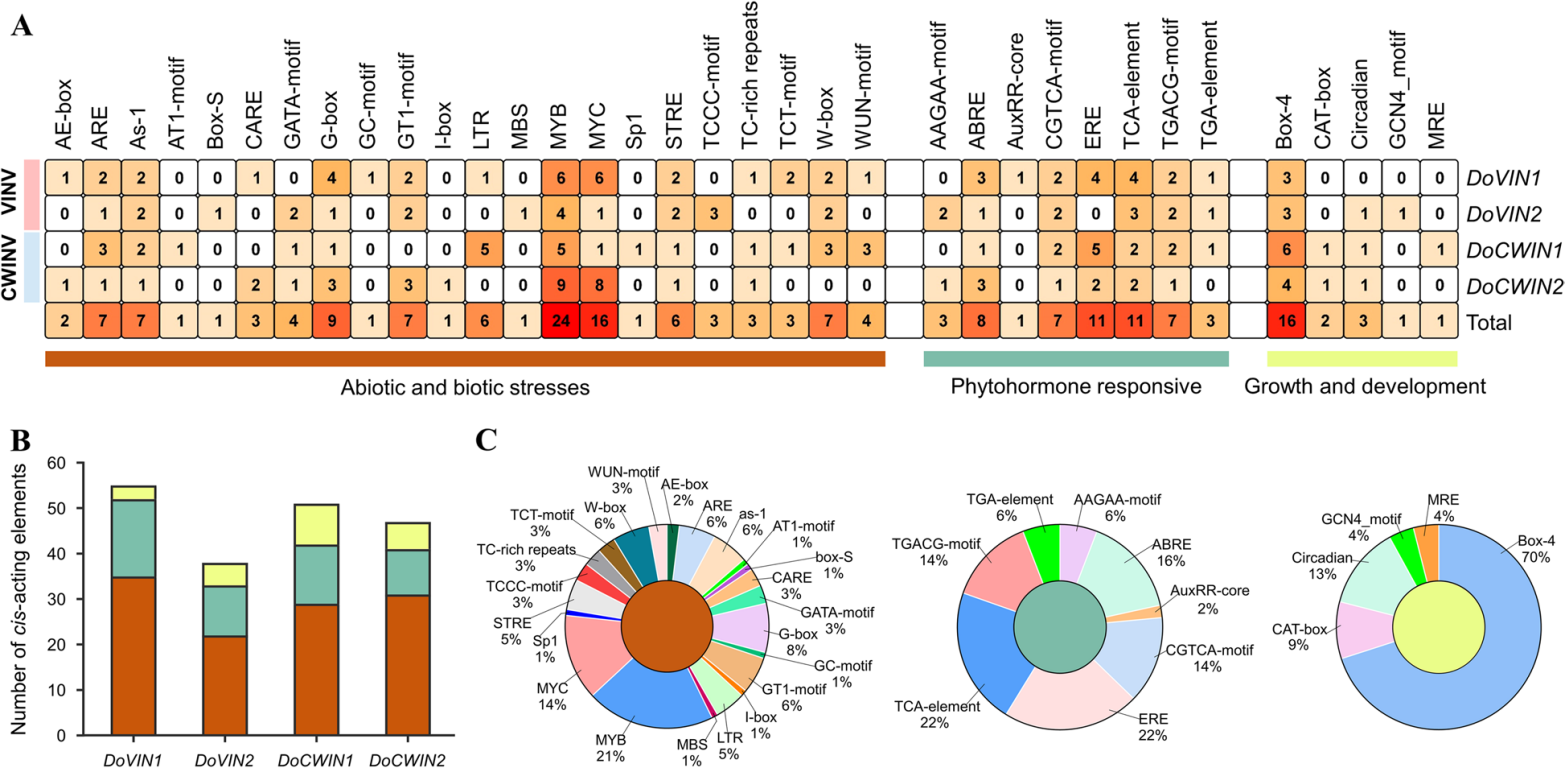
Investigation of *cis*-acting element numbers in *DoAINV* genes. **A** The different colors and numbers on the grid indicating the numbers of different promoter elements in four *DoAINV* genes. **B** The number of *cis*-acting elements belonging to each functional group in individual *DoAINV* promoter sequences. **C** Pie charts of different sizes indicating the ratio of each promoter element in each functional group, respectively

The *cis*-acting elements play a vital role in the regulatory networks that govern plant growth and development, as well as in determining the spatial–temporal and tissue-specific expression of genes. Utilizing the PlantCARE database, the *cis*-acting elements within the promoter regions of *DoAINV* genes were classified into three main groups: abiotic and biotic stress responses, phytohormone responses, and plant growth and development (Fig. 5). Notably, within all *cis*-acting element classes, those associated with abiotic and biotic stress responses were the most prevalent, followed by growth and development and phytohormone response classes, even within the *DoAINV* promoters (Fig. 5A, B). This implies that *AINV* genes may actively respond to abiotic stress and have the potential to enhance abiotic stress tolerance.

Within the abiotic and biotic stress responses category, various stresses-related elements were identified, including ARE (anaerobic induction), MBS (drought induction), LTR (low-temperature responses), TC-rich repeats (defense and stress responses), WUN-motif (wound responses), and other *cis*-acting elements (Fig. 5C). In the phytohormone responsive category, elements such as AuxRR-core and TGA-element associated with auxin response, ABRE and AAGAA-motif for the abscisic acid (ABA) response, and ERE for ethylene response were detected. Additionally, the TCA-element, linked to salicylic acid-responsive genes, was found in all *DoAINV* promoters. Notably, the most prevalent motifs were the TGACG-motif and CGTCA-motif, related to MeJA (Methyl Jasmonate) responsiveness, accounting for 28% of the scanned hormone-responsive motifs. Unusually, no *cis*-acting elements involved in gibberellin response were identified. Lastly, in the plant growth and development category, *cis*-acting elements were sparsely distributed in the promoter regions, including GCN4_motif for endosperm expression, CAT-box for meristem expression, Circadian for circadian control, and MRE and Box-4 for light responsiveness. Among these, Box-4 elements represented the majority, accounting for 70% of all elements. These findings imply that DoAINV genes may play diverse roles in plant growth and development.

### The organ-specific expression patterns of AINV genes in *D. officinale*

**Fig. 6:**
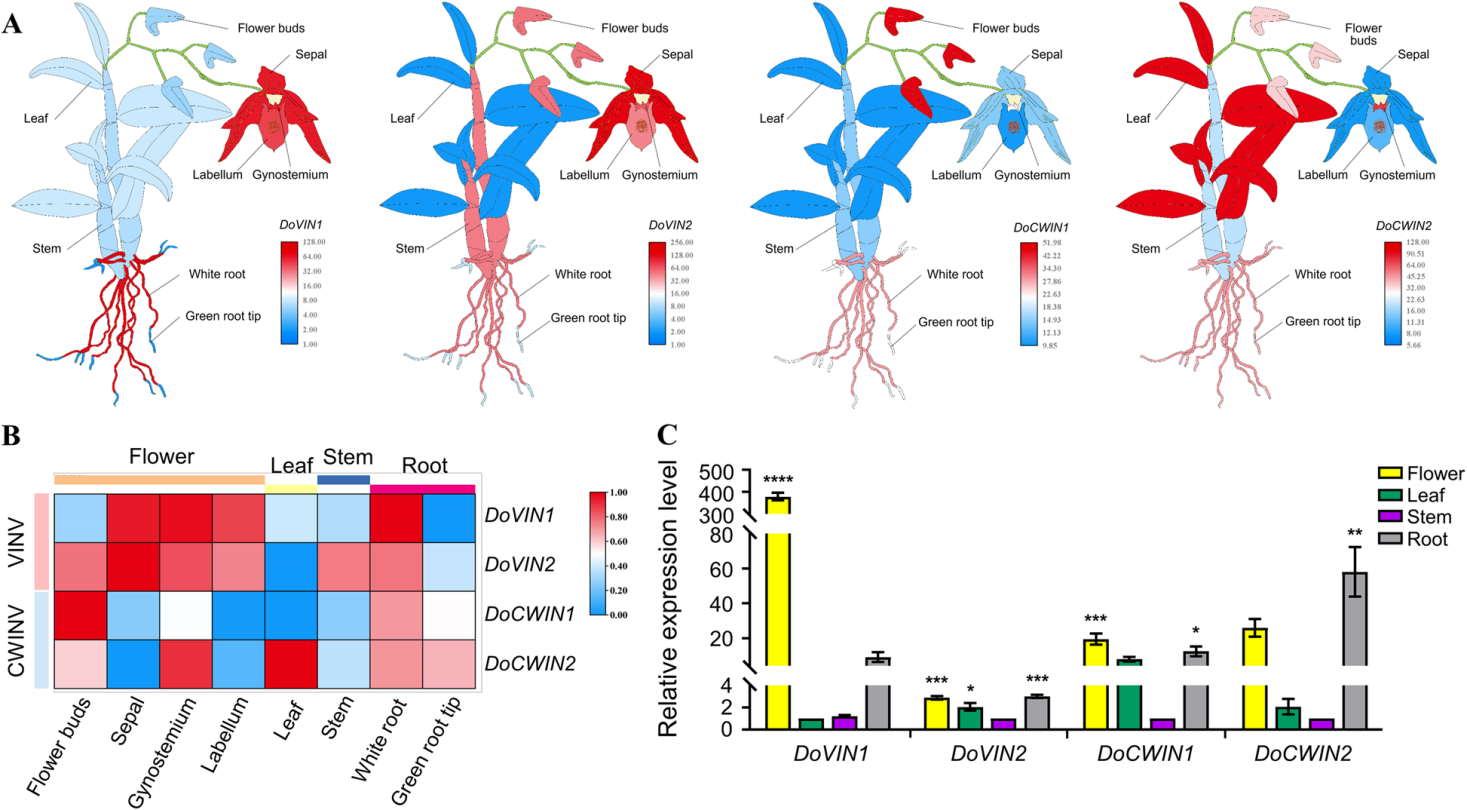
Tissue and organ expression pattern of *DoAINV* genes*.* **A** and **B** Organ-specific expression pattern of *DoAINV* genes in eight tissues: flower buds, speal, gynostemium, labellum, leaf, stem, white root, and green root tip. The heatmap was generated using TBtools software, based on log_2_ (FPKM). Blue and red indicat lower and higher levels of transcript abundance, respectively. **C** qRT-PCR validation of *DoAINVs* expression in different tissues obtained from six-month-old aseptic seedlings of the *D. officinale* DanXia cultivar. Floral organs were collected from one-year-old plants at the flowering stage. Transcripts were normalized to the expression of the actin gene. The mean ± standard deviations (SDs) of three biological replicates are presented, and the significant differences were analyzed by t-test. **** and *** significant difference represent *P* < 0.001, ** represent *P* < 0.01, and * represent* P* < 0.05

To further understand the potential functions of AINV gene familyin *D. officinale*, throughout its development, the expression profiling of *DoAINV* genes were performed by reanalyzing RNA-seq data from eight tissues: flower buds, sepal, gynostemium, labellum, leaf, stem, white root (equivalent to mature zone), and green root tip [39]. Based on the expression patterns, the four *DoAINV* genes exhibited distinct organ-specific expression (Fig. 6A). Within the VINV group, *DoVIN1* displayed high expression levels in sepal, gynostemium, labellum, and white root, while it was expressed at lower levels in green root tip, stem, leaf, and flower buds. *DoVIN2* showed high expression across all examined reproductive organs, including sepal, gynostemium, flower buds, and labellum, as well as in stem and white root. Among the CWINV group, flower buds exhibited the highest expression of *DoCWIN1*, followed by white root. *DoCWIN2* transcripts were most abundant in leaves and gynostemium, moderately expressed in flower buds and roots. These findings suggest that *DoAINV* genes might indirectly or directly participate in the development and formation of reproductive organs. In particular, each *DoAINV* gene displayed high expression in reproductive organs as well as other tissues such as leaf, stem, and root (Fig. 6B). The expression patterns of *DoAINV* genes were further evaluated and confirmed through qRT-PCR in selected organs of the *D. officinale* DanXia cultivar (Fig. 6C). This implies a diverse array of tissue expressions among genes within the same and different *DoAINV* clades, suggesting potential redundant, complementary, or alternate roles in plant growth and development.

### Regulation of of *D. officinale* AINV genes expression by abiotic stress

**Fig. 7:**
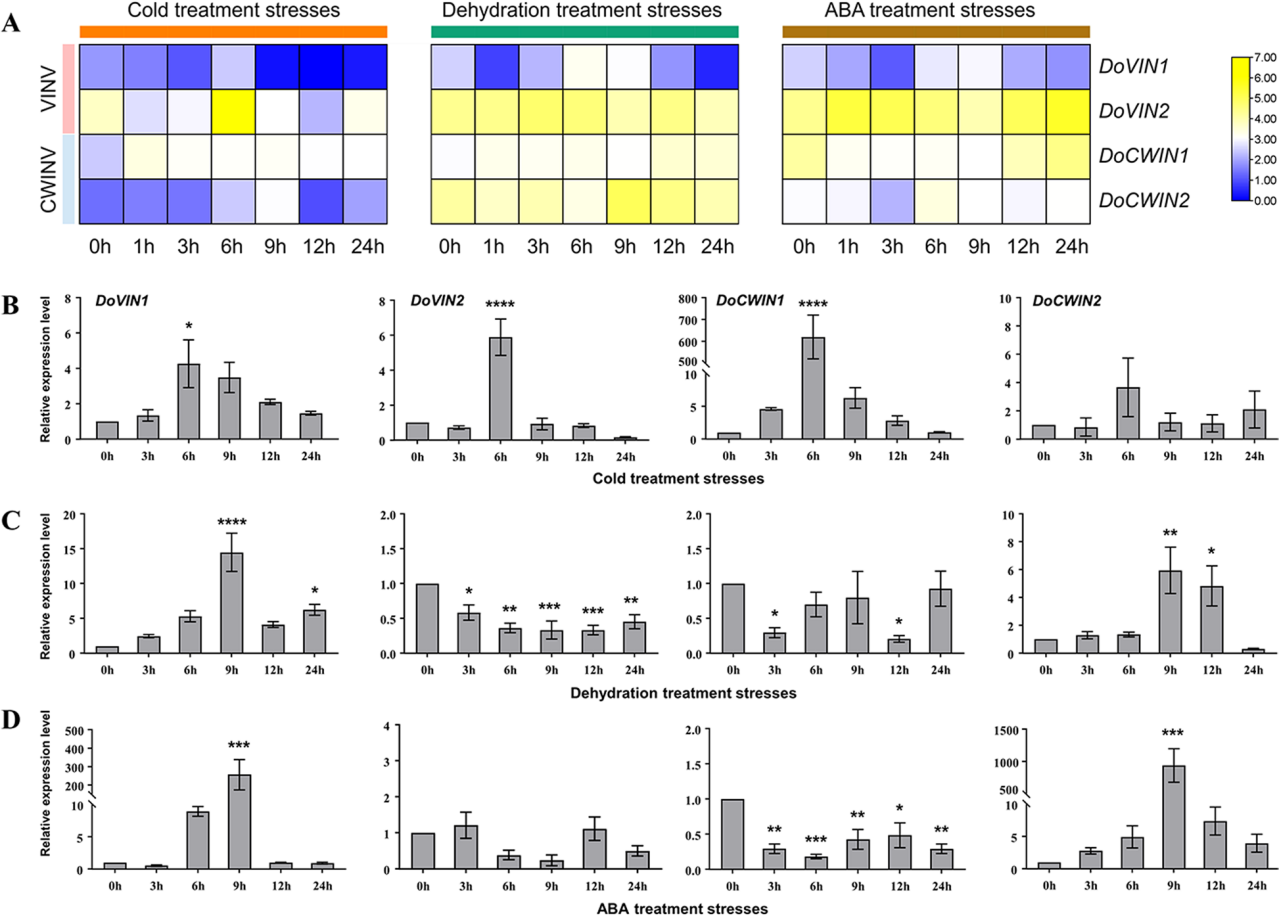
Expression patterns of *DoAINV* genes in response to abiotic stress. **A** Expression profiles of *DoAINV* genes in the *D. officinale* DanXia cultivar under cold, dehydration, and ABA treatment stresses. The heatmap was generated using TBtools software, based on the log_2_ (FPKM). Blue and red represent low and high levels of transcript abundance, respectively. **B**, **C** and **D** Expression levels of *DoAINV* genes in the *D. officinale* DanXia cultivar in response to cold, dehydration, and ABA treatment stresses, respectively, as determined by qRT-PCR experiments. The x-axis items, 0 h, 3 h, 6 h, 9 h, 12 h and 24 h, indicate different treatment times for cold stress (**B**), dehydration stress (**C**), and ABA stress (**D**), respectively. The y-axis represents the relative expression level. The values are the mean of three biological replicates, with SDs indicated by error bars. Significant differences were analyzed by t-test. **** and *** significant difference represents *P* < 0.001, ** Significant difference at *P* < 0.01, * significant difference (*P* < 0.05)

To explore the expression patterns of *DoAINV* genes in response to abiotic stress-related stimuli, the transcript levels of *DoAINV* genes in the DanXia cultivar under cold, dehydration, and ABA treatments were analyzed. The four *DoAINV* genes exhibited distinct expression patterns in response to the different treatments. Notably, all of them were up-regulated under cold treatment, reaching peak expression levels at 6 h or 9 h. Particularly, *DoVIN1* displayed up-regulated across all stress treatments, with the highest expression observed at 6 h or 9 h. Conversely, *DoVIN2* and *DoCWIN1* showed minimal changes during dehydration and ABA treatments, maintaining high expression levels relative to other two genes. *DoCWIN2* exhibited significant changes in expression patterns across the treatments (Fig. 7A). These findings confirm the potential involvement of *DoAINV* genes in abiotic stress responses, especially in cold resistance.

To further determine whether *DoAINV* genes were involved in abiotic stress resistance and the reliability of the transcriptome data, qRT-PCR was performed to measure the expression patterns and levels of *DoAINV* genes in the DanXia cultivar, also treated with cold, dehydration, and ABA stresses. Figure 7B to D illustrate that *DoVIN1* and *DoCWIN2* transcript abundances increased across all three treatments. Conversely, *DoVIN2* and *DoCWIN1* were significantly up-regulated under cold treatment but down-regulated or unaffected under the other treatments. Specifically, under cold stress, all four *DoAINV* genes were up-regulated, peaking at 6 h and gradually decreasing to untreated levels by 24 h (Fig. 7B). Notably, *DoCWIN1* exhibited particularly strong responsiveness, with transcript levels increasing almost 600-fold at 6 h of cold stress, indicating a crucial role in cold resistance for *D. officinale*. In dehydration treatment, *DoVIN1* and *DoCWIN2* expression levels were significantly up-regulated, reaching 15-fold and 6-fold increases, respectively, at 9 h (Fig. 7C). Despite variations in transcript levels among family members, *DoAINV* genes exhibited similar expression patterns under dehydration and ABA stress. For instance, *DoVIN1* and *DoCWIN2* transcript levels sharply increased at 9 h (over 200-fold and 1000-fold, respectively) after ABA treatment before rapidly decreasing (Fig. 7D). Similarly, *DoVIN2* and *DoCWIN1* shared similar expression patterns in response to dehydration stress and ABA treatment, suggesting potential roles of *DoVIN1* and *DoCWIN2* in dehydration and ABA stress responses in *D. officinale*.

**Fig. 8:**
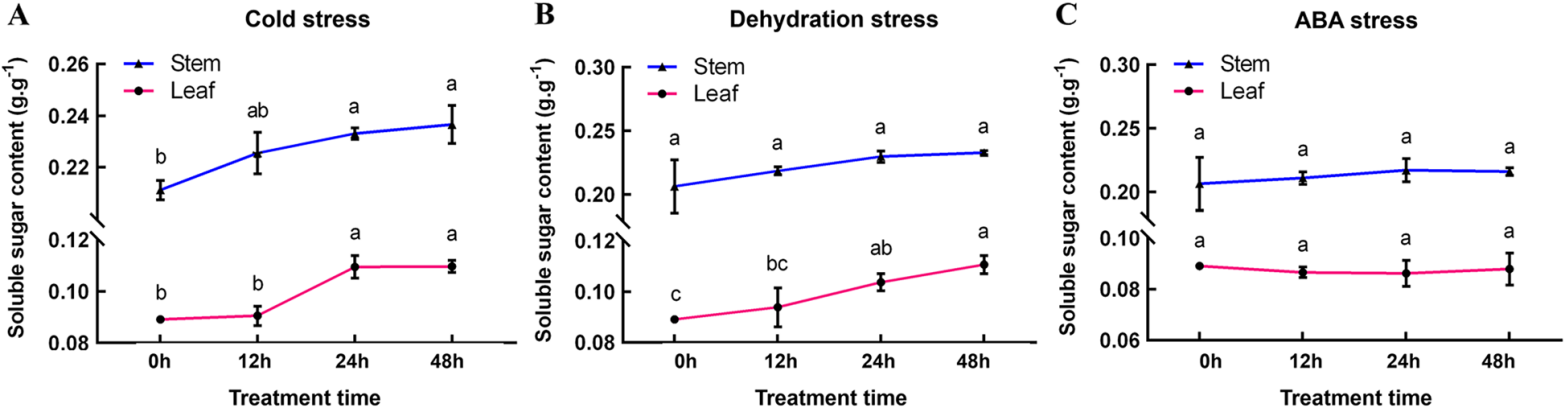
Soluble sugar content in *D. officinale* leaf and stem under different stress treatments. The x-axis represents different treatment times, including 0 h, 12 h, 24 h and 48 h, for cold (**A**), dehydration (**B**), and ABA (**C**) treatments, respectively. The y-axis represents the soluble sugar content. The values presented are the mean of three biological replicates, with standard deviations (SDs) indicated by error bars. Significant differences (*P* < 0.05) between treatments were analyzed using a one-way analysis of variance (ANOVA) in GraphPad Prism software and denoted by different letters above the bars

In order to investigate the correlation between the expression levels of the *DoAINV* genes and polysaccharide accumulation under cold, dehydration, and ABA treatments, soluble sugar content was measured in the leaves of *D. officinale* DanXia cultivar. The results revealed a significant increase in soluble sugar content at 24 h and 48 h compared to 0 h and 12 h under cold treatment (Fig. 8A) Similarly, soluble sugar content consistently increased during dehydration treatments in leaves compared to untreated control (Fig. 8B). Interestingly, the dramatic expression of *DoVIN1* and *DoCWIN2* genes under ABA treatment did not affect soluble sugar content accumulation in *D. officinale* (Fig. 8C). Additionally, it is worth mentioning that the stem of *D. officinale*, which has the highest polysaccharide content, is the tissue used for medicinal purposes [3, 7]. Therefore, the changing trend of soluble sugar content in the stem was also analyzed under different stress conditions. Overall, the results demonstrated that the soluble sugar content in the stem was higher compared to the leaves. Interestingly, the accumulation patterns in response to cold stress were almost the same in both leaves and stem (Fig. 8A). However, no significant differences were observed during the treatment periods of dehydration and ABA (Fig. 8B and C). In summary, these results highlight the varying expression of *DoAINV* genes in response to different abiotic stress, indicating diverse roles in abiotic stress responses.

### Interaction network analysis of AINV proteins in *D. officinale*

**Fig. 9:**
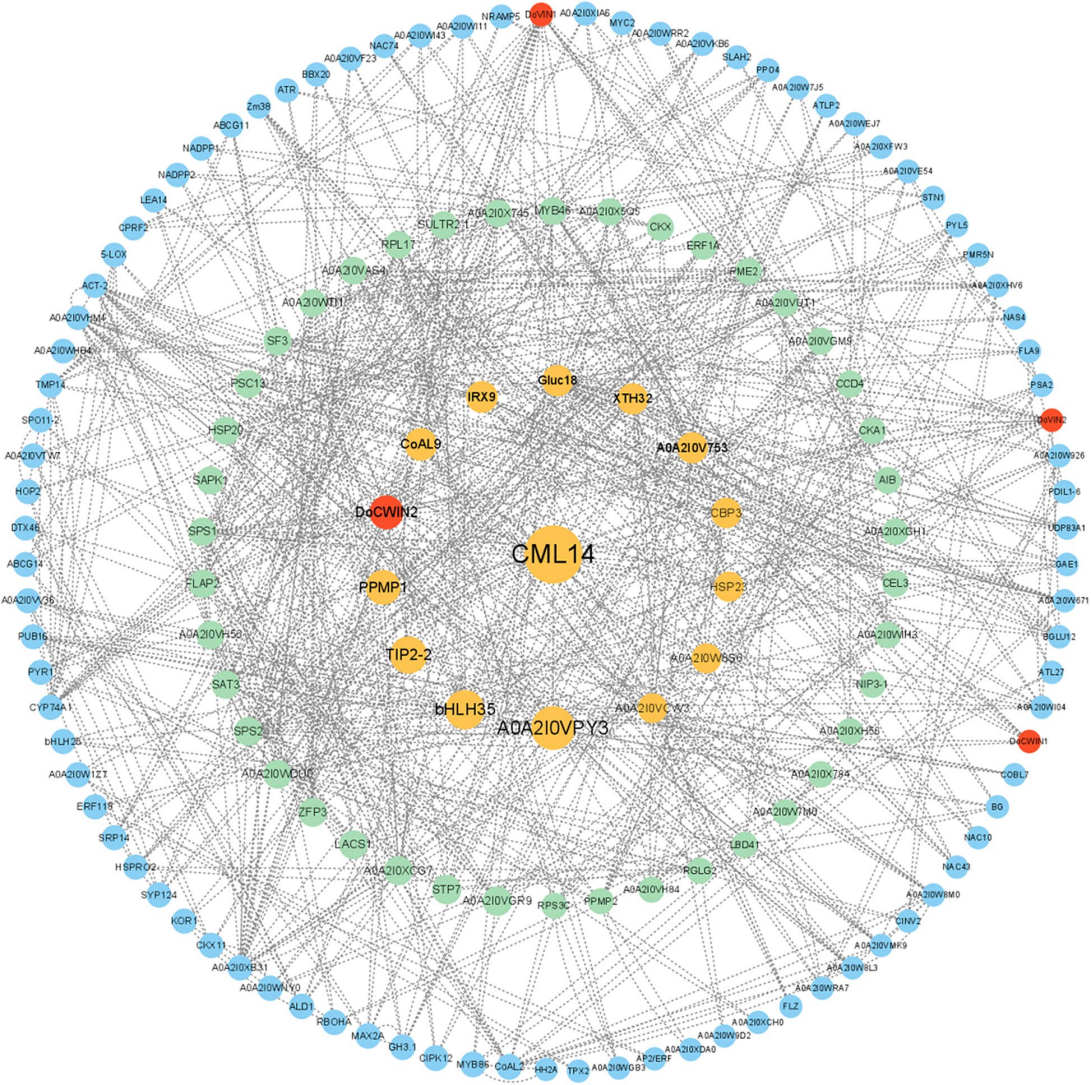
Protein–protein interaction (PPI) networks of DoAINV proteins. The network was generated based on the potential interaction data for 1,363 DEGs, extracted from the latest STRING database (STRING v11.5) and screened for transcriptomic data in cold stress treatment at 6 h compared to 0 h. Node size indicates its connectivity measured as node degree (i.e., the number of edges connecting the node); the bigger node means higher connectivity. Blue, green, and orange nodes represent different degrees of importance, with orange nodes indicating a Betweenness (BC) value > 1,000, green nodes presenting 1,000 > BC > 400, and blue nodes at BC < 400. Red nodes represent DoAINV family members. The edges (the connecting line) indicate the interaction between two genes, with the weight of the edges measuring their interaction strength. Thick edges denote strong interaction, whereas thin edges represent weak interaction

To gain deeper insights into the biological functions of DoAINVs in response to abiotic stress, possible protein–protein interaction (PPI) networks were further analyzed among DoAINV proteins and related proteins based on the potential interaction data for 1,363 differentially expressed genes (DEGs) using the new STRING 11.5 database. These DEGs were filtered based on a comparison of RNA-seq data from cold stress treatment at 6 h and untreated control (0 h). The genes harboring potential interaction relationships (Source-Target) are detailed in Supplementary Table S2 in Supplementary Material. The analysis, conducted using Cytoscape software (Version 3.9.1), identified a total of 147 nodes (genes) and 684 edges (interactions) in the PPI network (Fig. 9).

The PPI network, involved in cold stress signal transduction, revealed that DoAINV members had the strongest association with genes encoding calcium and calmodulin signaling network proteins (CML14, CBP3, and A0A2I0W7M0) (Fig. 9). Additionally, a second category of proteins closely interacting with DoAINVs included enzymes involved in carbohydrate synthesis and metabolism, such as IRX9, Gluc18, XTH32, SPS1, SPS2, and UDP83A1. Furthermore, the network comprised multiple receptor kinases, including CDPK gene families related to Ser/Thr protein kinases (STN7, CIPK12, and ATR), heat shock proteins (HSP20 and HSP23), and signal recognition proteins (SRP14, CPRF2, and A0A2I0V753). Notably, various types of transcription factors (TFs) related to adversity resistance and plant growth were also identified to interact with DoAINV proteins in the network, such as bHLH TFs (AIB, bHLH25, and bHLH35), MYB TFs (MYB46 and MYB86), ERF TFs (AP2/ERF, ERF1A, and ERF118) and NAC TFs (NAC10, NAC43, and NAC74). These results underscore the pivotal roles of DoAINV proteins in multiple biological functions, including stress response and plant growth.

## Discussion

### Characteristic features and evolutionary conservation among *D. officinale AINV* genes

Previous studies have elucidated variations in the number of AINV family members across different species, categorized into two subgroups: CWINV and VINV. For instance, *A. thaliana* harbors eight members, comprising two VINVs and six CWINVs [42], while rice possesses nine members, consisting of two VINVs and seven CWINVs [29], and poplar possesses eight members, encompassing three VINVs and five CWINVs [31]. In this study, all vacuolar INVs from base angiosperms, eudicots, and monocots were grouped into the VINV group based on phylogenetic analysis of AINVs from fourteen plant species. The VINV group was clearly distinguished from the cell-wall INV, grouped as the CWINV group. However, AINVs from early terrestrial plants remained clustered on the same branch, without division into VINV or CWINV subgroups (Fig. 2). Genome duplication events are frequent in higher plants. The salicoid duplication event, occurring approximately 65 million years ago, significantly contributed to the expansion of numerous multi-gene families through segmental duplication [43]. Evidence suggests that a genomic duplication event of a progenitor CWINV transpired in a common ancestor of pepper and tomato [44]. In Populus, estimation of Ks values revealed the ancient origins of CWINVs and indicated that the genome has undergone at least three rounds of genome-wide duplication [31]. These findings suggest that the origin of VINVs form CWINVs probably occurred in the early period of angiosperm formation, broadening previous deductions that the separation of AINVs predates the last common ancestor of dicots and monocots [45]. In the phylogenetic tree of AINVs from fourteen plant species, the VINV groups were categorized into four distinct categories: early terrestrial, base angiosperms, eudicots, and monocots. Interestingly, the number of CWINVs varied more among different plant species than VINVs. This discrepancy may be attributed to the diverse functions performed by members of these two subgroups. VINVs regulate the entry of sucrose into different utilization pathways [46], while CWINVs hydrolyze incoming translocated sucrose into glucose and fructose molecules, providing substrates for respiration and other metabolic processes [47]. Notably, the two VINVs of *D. officinale*, DoVIN1 and DoVIN2, were categorized within the monocotyledonous category. In the CWINV group, cell-wall INVs from fourteen plants exhibited similar evolutionary relationships, distributed in the four non-contiguous categories, following different branches. It is noteworthy that all CWINV members of monocotyledonous and eudicotyledonous plants were dispersed in two separate lineages in the phylogenetic tree, respectively. This observation may be linked to functional divergence of the CWINV subfamily during the evolution of monocotyledonous and dicotyledonous plants [44, 48]. These results also indicate that forerunners of AINVs in the CWINV group were more divergent than those in the VINV group.

The two CWINVs of *D. officinale* were distributed in two lineages of monocotyledonous plants, respectively. DoCWIN1was positioned on an earlier branch of monocotyledonous plants, leading to speculate that the ancestor of DoCWIN1 originated earlier than DoCWIN2. Interestingly, only two CWINVs were identified in *D. officinale*, significantly differing in number from other species, indicating no potential duplication events or gene functional redundancy in DoAINVs. This implies that the loss of CWINV genes occurred during the evolution of *D. officinale* in harsh environments such as cliffs, cold, and dry conditions. In such cases, the inability of *D. officinale* to form fruits, similar to rice, sweet sorghum, potato and other crops with high carbon sources and sugars throughout the development process, may ensue [29, 30, 38].

We also estimated the exon/intron structures of *DoAINV* genes to further understand the conservation and evolutionary relationship of *AINV* genes between dicot and monocot plants (Fig. 3). The integrated gene structure model in *DoAINV*s closely resembled that of *AINV* genes from *A. thaliana* and rice. A typical feature of the AINV gene family in plants is a mini second exon, located in the β-fructokinase motif (NDPNG), encoding only three amino acids [49]. In *D. officinale*, three out of four DoAINVs contain the conserved mini exon, except for *DoCWIN2*, suggesting that *DoCWIN2* experienced exons loss during evolution. This observation, not an isolated incident, aligns with *Os02g01590.1* of CWINV in rice; *PtrCWINV1* and *PtrCWINV2* in poplar [31]; and *ShCWINV3*, *ShCWINV8-1*, *ShCWINV9-1*, and *ShCWINV9-2* in sugarcane (*Saccharum officinarum*) [45]. Interestingly, despite *DoCWIN2* having the longest DNA sequence, even longer than any *AINV* genes in *A. thaliana* and rice, it contains only five exons. While motif comparison showed that the main motifs were conserved in all the *DoAINV* genes, suggesting gene structure variation was caused by exon splitting or intron length variation rather than pseudo-exonization. These findings further support the conclusions of Wang et al. [45]. Additionally, DoAINV proteins exhibited highly similar tertiary structural models, with all four forming a β-propeller module at the N-terminal domain and a β-sandwich module at the C-terminal domain (Fig. 4). This is consistent with reported AINVs in other plants [44, 50]. Overall, despite differences in genome length and exon/intron structure among *DoAINV* genes, they all exhibit typical conservative motifs and tertiary dimensional structures of AINVs, confirming that the gene organization is highly conserved within the plant AINV gene family.

### Sophisticated roles of *AINV* genes in *D. officinale*

The *AINV* gene family, serving as a pivotal enzyme in sucrose metabolism regulation, exhibits diverse members across species, with tissue-specific expression patterns observed in various organs and developmental stages. Previous studies have established the crucial role of AINV proteins in plant morphogenesis, growth, and development [4]. Analysis gene expression patterns in different tissues provides a reliable approach to studying the molecular functions of genes in various physiological processes. In our study, we found that *DoAINV* genes displayed tissue-specific expression patterns in *D. officinale*. Several *cis*-acting elements associated with flowering were identified in *DoAINV* promoter regions, indicating their potential role in reproduction organ formation and development by regulating sucrose metabolism (Fig. 5). Expression analysis revealed high expression of all *DoAINV* genes in specific floral organs of *D. officinal* (Fig. 6A and B), indicating their importance role in reproductive organ development. *DoVIN1* exhibited high expression in the white root, while *DoCWIN1* showed the highest expression in the tissue, suggesting these two genes potentially playing important physiological function in absorbing water and nutrient uptake. Conversely, *DoCWIN2* displayed the highest transcript abundance in the leaf, likely involved in phloem unloading in source organs. Additionally, *DoVIN2* exhibited high expression in stem, may play a vital role in regulating sucrose metabolism in vegetative organ. These results suggest diverse functions of *DoAINV* genes without functional redundancy, hinting at a functional collaboration among different DoAINV proteins in polysaccharide biosynthesis.

In addition to plant development, AINV proteins are associated with plant signal transduction and responses to various environmental stress [51, 52]. In our study, a variety of frequently occurring *cis*-acting elements was identified in the promoter regions of *DoAINVs*, suggesting their involvement in responding to both biotic and abiotic stress. Under cold, dehydration, and ABA treatments, the expression levels of *DoVIN1* and *DoCWIN2* were up-regulated,indicating their predominant role in responding to abiotic stress. *DoVIN2* and *DoCWIN1* were significantly up-regulated in response to cold stress, while their expression was down-regulated or unaffected after dehydration and ABA treatments. Similar expression response of *AINV* genes have been observed in sugarcane [45], suggesting conserved regulatory mechanisms across plant species.

Polysaccharide accumulation and sucrose metabolism in higher plant are often influenced by growth conditions and exogenous hormones, leading to changes in sugar contents and various physiological functions to resist environmental stress [53]. We examined soluble sugar content under cold, dehydration, and ABA treatments and observed that it was affected by cold and dehydration treatment but remained unchanged in response to ABA treatment (Fig. 8). The increase in soluble sugar content in *D. officinale* in response to cold stress and dehydration treatment likely resulted from up-regulation of *AINV* gene expression to enhance protein activity and cleaved more sucrose into glucose and/or fructose to maintain osmotic homeostasis and enhance stress resistance [46, 54]. All four *DoAINV* genes were up-regulated (especially *DoCWIN1*) under cold stress (Fig. 7B), consistent with findings in populus [31] and tulip [55], suggesting induction of both *VINVs* and *CWINVs* to ensure sucrose accumulation and promote unloading for glucose or fructose production to maintain osmotic homeostasis and improve cold tolerance. However, *DoVIN1* and *DoCWIN2* of the examined genes were up-regulated, and *DoVIN2* and *DoCWIN1* were down-regulated under dehydration treatment (Fig. 7C), aligns with the expression patterns of *AINV* genes in sugarcane [45]. This suggests that the raised *AINVs* levels may be necessary to enhance the ability to stabilize osmotic homeostasis, while the down-regulated of *AINV* genes could be due to the need for receiving and integrating sugar modulation signals to block downstream metabolism and adapt to dehydration stress. Thus, there may be difference in the molecular response mechanism between cold and dehydration stress. Under dehydration stress, *DoVIN1* and *DoCWIN2* contributed the most to polysaccharide synthesis. Noteworthy, cold and dehydration stress may regulate gene expression despite decreasing actual protein activity in the plant. In our study, we compared soluble sugar content between the stem and leaf of *D. officinale* and observed consistent accumulation patterns in both tissues. Remarkably, the soluble sugar content in both leaf and stem, during cold stress and dehydration treatment was increased compared with untreated control (Fig. 8), especially under dehydration treatment, significant differences were observed in the soluble sugar content of the leaf at different time points (Fig. 8B). This suggests a response mechanism to mitigate damage form environmental stressors. The increase in AINV protein may serve to enhance actual protein activity under these conditions, accelerating sucrose hydrolysis and leading to polysaccharides accumulation to maintain osmotic homeostasis, protect membrane stability, and reduce or prevent damage to plant, including but not limited to leaves and stems.

Furthermore, the induction of *AINV* gene expression by ABA, a crucial phytohormone, has been observed in various plant species, including green bamboo (*Bambusa oldhamii*) [56]. Consistent with these findings, our study showed that after ABA treatment, two DoAINV genes (*DoVIN1* and *DoCWIN2*) were significantly up-regulated, while the other two genes (*DoVIN2* and *DoCWIN1)* were down-regulated (Fig. 7D). This expression pattern mirrors that observed under dehydration stress (Fig. 7C), indicating that *DoVIN1* and *DoCWIN2* may play a major role in the response of the entire AINV gene family to both dehydration stress and ABA treatment. Interestingly, although the increased transcript abundance of *DoVIN1* and *DoCWIN2* genes did not significantly alter the soluble sugar content of *D. officinale* during ABA treatment (Fig. 8C), this could be explained by this could be explained by the multifaceted functions of AINVs. Besides their role in sucrose degradation and sugar concentration maintenance, AINVs may also act as signaling molecules in modulating different signal transduction pathways and protecting cellular structural integrity from damage. These results underscore the diverse functions of *AINV* genes in *D. officinale* and provided evidence that plant AINV members can participate in abiotic stress responses. The gene expression patterns, changes in soluble sugar content, and PPI networks under abiotic stress conditions contribute to a deeper understanding of the interactive regulatory network of AINV genes and polysaccharide biosynthesis pathways.

## Conclusions

In this study, four *D. officinale* AINV genes were identified and characterized bioinformatically. The *DoAINV* family members were found to share two conserved domains and were classified into two subfamilies based on the cellular locations and phylogenetic analysis. Furthermore, the functional characteristics of the DoAINV domains were identified. The expression of the four *DoAINV* genes was observed to be up-regulated under cold stress, aiming to protect cellular structural integrity from damage. Similarly, under dehydration stress and ABA treatment, the expression of *DoVIN1* and *DoCWIN2* was also up-regulated to meet the requirement for cleaving more sucrose. Conversely, the expression of *DoVIN2* and *DoCWIN1* could be down-regulated to preserve sucrose homeostasis. Specifically, the expression of *DoAINV* genes led to an increase in soluble sugar content of *D. officinale* in response to cold and dehydration stresses. Based on our experimental results, it is speculated that DoAINVs play a significant role in regulating sucrose metabolism and polysaccharide accumulation, as well as serving as signaling molecules to modulate ABA signal transduction pathways. In conclusion, our study provides insightful perspectives on the function of *DoAINV* genes in alleviating stress and polysaccharide biosynthesis.

## Methods

### Plant materials, growth conditions and treatments

The *D. officinale* used in this work were sourced from the DanXia cultivar, cultivated at the Engineering Technology Development Center of *Dendrobium* herb in Shaoguan, China. Following surface disinfection of *D. officinale* capsules, the seeds were sown in half-strength Murashige and Skoog (1/2 MS) medium (Sigma-Aldrich, St. Louis, MO, USA), supplemented with20% potato flour, and subjected to sterile growth conditions at a temperature of 25 ± 1 ℃, with a light/dark cycle of 12/12 h, and a relative humidity of 60–70% within a growth chamber at Shaoguan University (Shaoguan, China). The aseptic seedlings underwent subculture every two months until the stems were 5–6 cm, approximately six months post-germination. Then young seedlings were selected for various treatments. For each treatment, six leaves from six separate seedlings were pooled to form one sample, and all experiments were performed in triplicate.

For exogenous ABA treatment, seedlings were cultured in liquid 1/2 MS supplemented with 100 μM ABA for 2 days. For dehydration stress treatment, seedlings were cultured in 1/2 MS supplemented with 20% polyethylene glycol (PEG) 6000 for 2 days. Seedlings cultured in 1/2 MS served as control. For cold stress treatment, seedlings were moved to a growth chamber set at a temperature of 4 ± 1 ℃ for 2 days. Untreated seedlings were used as controls. Subsequently, all treated leaves were harvested at 0-, 1-, 3-, 6-, 9-, 12-, 24- and 48-h post-treatment, flash-frozen in liquid nitrogen, and stored at -80 ℃ for further expression analysis.

Moreover, leaf, stem, root, and flower samples were collected from *D. officinale* plants during the flowering stage grown under normal conditions. These samples were also flash-frozen in liquid nitrogen and stored at -80 ℃ for further expression analysis. Three biological replicates were collected for each sample.

### Identification of the AINV family genes from *D. officinale* genome

The whole-genome sequence of *D. officinale* were obtained from the National Centre for Biotechnology Information (NCBI, http://www.ncbi.nlm.nih.gov/). Subsequently, the HMM profiles corresponding to the glycosyl hydrolases family 32 N-terminal domain (PF00251) and 32 C-terminal domain (PF08244), which represent conserved domain of the AINV gene family, were retrieved from Pfam (http://pfam.xfam.org/, 35.0). These HMM profiles were then used to identify all AINV proteins encoded in the *D. officinale* genome. Additionally, to avoid any non-specific sequences located outside the AINV cluster, sequences of eight AINVs from *A. thaliana* [26] and nine AINVs from rice [29] were emplyed as queries in iterative BLAST searches against the *D. officinale* database using default parameters. Finally, all candidate DoAINV genes were subjected manual verification using the Conserved Domains Database (http://www.ncbi.nlm.nih.gov/cdd) to confirm the completeness of all core domains.

### Protein properties and sequence analyses

The molecular weights and isoelectric points of the DoAINVs were predicted using the Expasy database (http://expasy.org/). Motifs were identified using the MEME (Multiple Em for Motif Elicitation) software (http://meme.nbcr.net/meme/cgi-bin/meme.cgi) and visualized using the “visualize motif pattern” function of the ToolKit for Biologists (TBtools) software (https://github.com/CJ-Chen/TBtools) [57]. Motifs identified by MEME were further queried in the InterPro database (http://www.ebi.ac.uk/interpro/). The gene structure of *DoAINVs* were determined using the Gene Structure Display Server (http://gsds.cbi.pku.edu.cn/, 2.0) by aligning complementary DNA (cDNA) sequences with their corresponding genomic DNA sequences. Protein domain were annotated using the PROSITE bioinformatics tool (https://prosite.expasy.org/cgi-bin/prosite/mydomains/). Secondary and tertiary structures were predicted using SOPMA (https://npsa-prabi.ibcp.fr/cgi-bin/npsa_automat.pl?page=npsa_sopma.html) and the ExPaSy Swiss-Model online software (http://swissmodel.expasy.org), respectively. Transmembrane helices and signal peptides were predicted using the TMHMM program (https://services.healthtech.dtu.dk/service.php?TMHMM-2.0) and SignalP 5.0 Server (https://services.healthtech.dtu.dk/service.php?SignalP-5.0), respectively. Phosphorylation sites were predicted using the NetPhos 3.1 Servers (https://services.healthtech.dtu.dk/service.php?NetPhos-3.1). The subcellular localization were predicted using the Cell-PLoc 2.0 (http://www.csbio.sjtu.edu.cn/bioinf/Cell-PLoc-2/) online tools.

### *Cis*-acting element and phylogenetic analyses

The promoter sequences, spanning 2.0 kb upstream of the start codon, were extracted from *D. officinale* genomic sequences. The *cis*-acting elements within the *DoAINV* promoters were analyzed using the PlantCARE database (http://bioinformatics.psb.ugent.be/webtools/plantcare/html/). The identified promoter elements, relevant to phytohormones, environmental response, and adversity adaptation, were categorized and visualized using the Simple BioSequence Viewer function of TBtools. DNAMAN (version 6.0) were used for multiple alignments of the identified DoAINVs nucleotide and amino acid sequences. The phylogenetic tree of AINV amino acids form different species was constructed using MEGA 11 and Clustal X (version 2.0) with the neighbor-joining (NJ) algorithm, and bootstrap testing was performed with 1, 000 replicates [58, 59].

### Transcriptomic analysis

The sequencing procedures were completed by BGI Tech Co., Ltd. (Shenzhen, China), including RNA extraction, library construction, and sequencing. Illumina Hiseq™ 2500/Miseq™ (Illumina, USA) served as the sequencing platform. To ensure data quality, adapter sequences and low-quality reads were eliminated using the FASTX toolkit (http://hannonlab.cshl.edu/fastx_toolkit/) and FastQC program (http://www.bioinformatics.babraham.ac.uk/projects/fastqc/), respectively. Bioinformatic analyses of the raw sequencing data were conducted by Yanke Gene (Gangzhou, China). Expression levels were quantified and normalized as fragments per kilobase per million mapped fragments (FPKM). The DEGseq R package (version 1.12.0) was used to identify significantly DEGs (Log2-based fold-changes > 1; Log2-based fold-changes <  − 1) in response to treatment.

### In silico expression profiling of *D. officinale* AINV genes in different tissues

For tissue expression profiling of *DoAINV* genes, raw RNA-seq reads from various tissues including flower buds (SRR4431603), gynostemium (SRR4431596), labellum (SRR4431602), sepal (SRR4431597), leaf (SRR4431601), stem (SRR4431600), white part of root (SRR4431598) and green root tip (SRR4431599) were obtained from the NCBI Sequence Read Archive (SRA) [39]. Subsequently, all samples’ reads were aligned to the NCBI *Dendrobium* reference genome using the HISAT package [60]. FPKM were calculated for mRNA expression quantification using StringTie [61]. The resulting heatmap was generated using the eFP Browser feature of TBtools.

### RNA extraction, cDNA synthesis and qRT-PCR

Total RNA was isolated from the leaves using the MiniBEST Plant RNA Extraction Kit (9769, TaKaRa, Japan). Subsequently, first-strand cDNA synthesis was performed using the PrimeScript™ RT Master Mix (RR036A, TaKaRa, Japan). A PCR reaction mixture (20 μL) was prepared according to the manual of the TB Green® Premix Ex *Taq*™ (Tli RNaseH Plus) Kit (RR820A, TaKaRa, Japan), in accordance with the manufacturer’s instructions. The reaction was conducted using the BIO-RAD CFX Connect™ Real-time System (BIORAD, USA). The *DoACTIN* gene was employed as an internal control [62]. Each experiment was replicated in triplicate, and three biological replicates were conducted. The primers used in the qRT-PCR experiments are listed in Table S3. Relative expression levels were calculated according to the Normalized Expression method (2^*−△△CT*^ method) [63].

### Measurement of total soluble sugar content

The total soluble sugar was extracted from the leaf and stem of *D. officinale* DanXia cultivar seedlings, respectively, using the water extraction and alcohol precipitation method. Then soluble sugar content was measured utilizing the phenol–sulfuric acid method, employing ultraviolet spectroscopy with anhydrous glucose as the reference substance at a wavelength 483 nm wavelength. The content was calculated through a standard curve of anhydrous glucose, which exhibited excellent linearity (*r* = 0.999 3), based on the regression equation y = 3.5696x + 0.0065.

### Protein–protein interaction network construction and visualization

The potential interaction data between DoAINVs and related proteins under cold stress treatment were extracted from the latest STRING v11.5 available database (https://cn.string-db.org/) [64]. Subsequently, the interactions between the genes was constructed using Cytoscape v3.9.1 software (https://cytoscape.org/) for visualization purposes. The Cytoscape plugin within CytoNCA was utilized to identify the key targets [65].

### Statistical analysis

All data were subjected to analysis using either a one-way analysis of variance (ANOVA) or a Student’s t-test, with a significance level set at 0.05, utilizing GraphPad Prism v9.5 (GraphPad Software, Inc., Chicago, USA, www.graphpad.com). The analyses were performed based on three biological replicates, and the values shown in the figures represent the average values derived from three replicates. Sample variability is depicted as the standard deviations (SDs) of the mean.

### Supplementary Information

Supplementary Material 1.

## Data Availability

The datasets supporting our conclusions in this study are provided in the manuscript and additional files.The genome sequence of *D. officinale* was downloaded from the NCBI database. AINV protein sequences from *A. thaliana* and rice were retrieved from The *Arabidopsis* Information Resource (TAIR, http://www.arabidopsis.org/) and the Rice Genome Annotation Project (RGAP, http://rice.plantbiology.msu.edu/) databases, respectively. Tissue expression profiling analysis used previously published raw sequence data of *D. officinale* obtained from NCBI-BioProject PRJNA348403. The generated sequencing data in this study have been deposited in NCBI’s SRA database. The raw sequence data of *D. officinale* in response to abiotic stress are available in NCBI-BioProject database under the accession number PRJNA943399, PRJNA949800, and PRJNA949802, respectively.

## References

[1] Zhu S, Niu Z, Xue Q, Wang H, Xie X, Ding X (2018). Accurate authentication of *Dendrobium*
*officinale* and its closely related species by comparative analysis of complete plastomes. Acta Pharm Sin B.

[2] Li L, Jiang Y, Liu Y, Niu Z, Xue Q, Liu W (2020). The large single-copy (LSC) region functions as a highly effective and efficient molecular marker for accurate authentication of medicinal *Dendrobium* species. Acta Pharm Sin B.

[3] Ng TB, Liu J, Wong JH, Ye X, Wing Sze SC, Tong Y (2012). Review of research on *Dendrobium*, a prized folk medicine. Appl Microbiol Biotechnol.

[4] Wang Y, Liu A (2020). Genomic characterization and expression analysis of basic Helix-Loop-Helix (bHLH) family genes in traditional chinese herb *Dendrobium*
*officinale*. Plants.

[5] Wang H, Dong Z, Chen J, Wang M, Ding Y, Xue Q (2022). Genome-wide identification and expression analysis of the Hsp20, Hsp70 and Hsp90 gene family in *Dendrobium*
*officinale*. Front Plant Sci.

[6] Yan L, Wang X, Liu H, Tian Y, Lian J, Yang R (2015). The genome of *Dendrobium*
*officinale* Illuminates the biology of the Important traditional chinese orchid herb. Mol Plant.

[7] Tang H, Zhao T, Sheng Y, Zheng T, Fu L, Zhang Y (2017). *Dendrobium*
*officinale* Kimura et Migo: a review on its ethnopharmacology, phytochemistry, pharmacology, and industrialization. Evid Based Complement Alternat Med.

[8] Shen C, Guo H, Chen H, Shi Y, Meng Y, Lu J (2017). Identification and analysis of genes associated with the synthesis of bioactive constituents in *Dendrobium*
*officinale* using RNA-Seq. Sci Rep.

[9] Xing S, Zhang X, Ke H, Lin J, Huang Y, Wei G (2018). Physicochemical properties of polysaccharides from *Dendrobium*
*officinale* by fractional precipitation and their preliminary antioxidant and anti-HepG2 cells activities in vitro. Chem Cent J.

[10] Liang J, Wu Y, Yuan H, Yang Y, Xiong Q, Liang C (2019). *Dendrobium*
*officinale* polysaccharides attenuate learning and memory disabilities via anti-oxidant and anti-inflammatory actions. Int J Biol Macromol.

[11] Zhong C, Tian W, Chen H, Yang Y, Xu Y, Chen Y (2022). Structural characterization and immunoregulatory activity of polysaccharides from *Dendrobium*
*officinale* leaves. J Food Biochem.

[12] Liu Y, Yang L, Zhang Y, Liu X, Wu Z, Gilbert RG (2020). *Dendrobium*
*officinale* polysaccharide ameliorates diabetic hepatic glucose metabolism via glucagon-mediated signaling pathways and modifying liver-glycogen structure. J Ethnopharmacol.

[13] Kuang M, Li J, Yang X, Yang L, Xu J, Yan S (2020). Structural characterization and hypoglycemic effect via stimulating glucagon-like peptide-1 secretion of two polysaccharides from *Dendrobium*
*officinale*. Carbohydr Polym.

[14] Li J, Li S, Huang D, Zhao X, Cai G (2011). Advances in the of resources, constituents and pharmacological effects of *Dendrobium*
*officinale*. Sci Technol Rev.

[15] Huang K, Li Y, Tao S, Wei G, Huang Y, Chen D (2016). Purification, characterization and biological activity of polysaccharides from *Dendrobium*
*officinale*. Mol Basel Switz.

[16] Zhang B, Tolstikov V, Turnbull C, Hicks LM, Fiehn O (2010). Divergent metabolome and proteome suggest functional independence of dual phloem transport systems in cucurbits. Proc Natl Acad Sci U S A.

[17] Li Q, Li B, Guo S (2016). Advance in molecular biology of *Dendrobium* (Orchidaceae). China J Chin Mater Medica.

[18] Li M, Chen T, Gao T, Miao Z, Jiang A, Shi L (2015). UDP-glucose pyrophosphorylase influences polysaccharide synthesis, cell wall components, and hyphal branching in *Ganoderma*
*lucidum* via regulation of the balance between glucose-1-phosphate and UDP-glucose. Fungal Genet Biol FG B.

[19] Wang X, Wang S, Xue Y, Ren X, Xue J, Zhang X (2020). Defoliation, not gibberellin, induces tree peony autumn reflowering regulated by carbon allocation and metabolism in buds and leaves. Plant Physiol Biochem PPB.

[20] Zhou T, Hao G, Yang Y, Liu H, Yang M, Zhao Y (2019). *Sicwinv1*, a cell wall invertase from sesame, is involved in Anther development. J Plant Growth Regul.

[21] Chen B, Wang X, Lv J, Ge M, Qiao K, Chen Q (2021). GhN/AINV13 positively regulates cotton stress tolerance by interacting with the 14-3-3 protein. Genomics.

[22] Wang X, Zheng L, Lin H, Yu F, Sun L, Li L (2017). Grape hexokinases are involved in the expression regulation of sucrose synthase- and cell wall invertase-encoding genes by glucose and ABA. Plant Mol Biol.

[23] Yan W, Wu X, Li Y, Liu G, Cui Z, Jiang T (2019). Cell wall invertase 3 affects cassava productivity via regulating sugar allocation from source to sink. Front Plant Sci.

[24] Roitsch T, González M (2004). Function and regulation of plant invertases: sweet sensations. Trends Plant Sci.

[25] Ruan Y, Jin Y, Yang Y, Li G, Boyer JS (2010). Sugar input, metabolism, and signaling mediated by invertase: roles in development, yield potential, and response to drought and heat. Mol Plant.

[26] Sherson SM, Alford HL, Forbes SM, Wallace G, Smith SM (2003). Roles of cell-wall invertases and monosaccharide transporters in the growth and development of *Arabidopsis*. J Exp Bot.

[27] Sturm A (1996). Molecular characterization and functional analysis of sucrose-cleaving enzymes in carrot (*Daucus*
*carota* L.). J Exp Bot.

[28] Haouazine-Takvorian N, Tymowska-Lalanne Z, Takvorian A, Tregear J, Lejeune B, Lecharny A (1997). Characterization of two members of the *Arabidopsis*
*thaliana* gene family, *Atβfruct3* and *Atβfruct4*, coding for vacuolar invertases. Gene.

[29] Ji X, Van den Ende W, Van Laere A, Cheng S, Bennett J (2005). Structure, evolution, and expression of the two invertase gene families of rice. J Mol Evol.

[30] Liu Y, Nie YD, Han FX, Zhao XN, Dun BQ, Lu M (2014). Allelic variation of a soluble acid invertase gene (*SAI-1*) and development of a functional marker in sweet sorghum [*Sorghum*
*bicolor* (L.) Moench]. Mol Breed.

[31] Chen Z, Gao K, Su X, Rao P, An X (2015). Genome-wide identification of the invertase gene family in populus. PLoS ONE.

[32] Liu Y, Dun B, Zhao X, Yue M, Lu M, Li G (2013). Correlation analysis between the key enzymes activies and sugar content in sweet sorghum (*Sorghum*
*bicolor* L. Moench) stems at physiological maturity state. Aust J Crop Sci.

[33] Wang L, Ruan Y (2016). Critical roles of vacuolarinvertase in floral organ development and male and female fertilities are revealed through characterization of *GhVIN1*-RNAi cotton plants. Plant Physiol.

[34] Qian W, Yue C, Wang Y, Cao H, Li N, Wang L (2016). Identification of the invertase gene family (INVs) in tea plant and their expression analysis under abiotic stress. Plant Cell Rep.

[35] Godt DE, Roitsch T (1997). Regulation and tissue-specific distribution of mRNAs for three extracellular invertase isoenzymes of tomato suggests an important function in establishing and maintaining sink metabolism. Plant Physiol.

[36] Shen L, Yao Y, He H, Qin Y, Liu Z, Liu W (2018). Genome-wide identification, expression, and functional analysis of the alkaline/neutral invertase gene family in pepper. Int J Mol Sci.

[37] Yao Y, Geng M, Wu X, Liu J, Li R, Hu X (2015). Genome-wide identification, expression, and activity analysis of alkaline/neutral invertase gene family from cassava (*Manihot*
*esculenta* Crantz). Plant Mol Biol Report.

[38] Liu X, Zhang C, Ou Y, Lin Y, Song B, Xie C (2011). Systematic analysis of potato acid invertase genes reveals that a cold-responsive member, *StvacINV1*, regulates cold-induced sweetening of tubers. Mol Genet Genomics MGG.

[39] Zhang G, Xu Q, Bian C, Tsai W, Yeh C, Liu K (2016). The *Dendrobium*
*catenatum* Lindl. genome sequence provides insights into polysaccharide synthase, floral development and adaptive evolution. Sci Rep.

[40] Nonis A, Ruperti B, Pierasco A, Canaguier A, Adam-Blondon A-F, Di Gaspero G (2008). Neutral invertases in grapevine and comparative analysis with *Arabidopsis*, poplar and rice. Planta.

[41] Shao Z, Xue J, Wu P, Zhang Y, Wu Y, Hang Y (2016). Large-Scale analyses of angiosperm nucleotide-binding site-leucine-rich repeat genes reveal three anciently diverged classes with distinct evolutionary patterns. Plant Physiol.

[42] Verhaest M, Lammens W, Le Roy K, De Coninck B, De Ranter CJ, Van Laere A (2006). X-ray diffraction structure of a cell-wall invertase from *Arabidopsis*
*thaliana*. Acta Crystallogr D Biol Crystallogr.

[43] Zuo R, Hu R, Chai G, Xu M, Qi G, Kong Y (2013). Genome-wide identification, classification, and expression analysis of CDPK and its closely related gene families in poplar (*Populus*
*trichocarpa*). Mol Biol Rep.

[44] Shen L, Qin Y, Qi Z, Niu Y, Liu Z, Liu W-X (2018). Genome-Wide analysis, expression profile, and characterization of the acid invertase fene gamily in pepper. Int J Mol Sci.

[45] Wang L, Zheng Y, Ding S, Zhang Q, Chen Y, Zhang J (2017). Molecular cloning, structure, phylogeny and expression analysis of the invertase gene family in sugarcane. BMC Plant Biol.

[46] Invertases SA (1999). Primary structures, functions, and roles in plant development and sucrose partitioning. Plant Physiol.

[47] Kulshrestha S, Tyagi P, Sindhi V, Yadavilli KS (2013). Invertase and its applications – A brief review. J Pharm Res.

[48] Zhang J, Wu Z, Hu F, Liu L, Huang X, Zhao J (2018). Aberrant seed development in *Litchi*
*chinensis* is associated with the impaired expression of cell wall invertase genes. Hortic Res.

[49] Bocock PN, Morse AM, Dervinis C, Davis JM (2008). Evolution and diversity of invertase genes in *Populus*
*trichocarpa*. Planta.

[50] Yao Y, Geng M, Wu X, Liu J, Li R, Hu X (2014). Genome-wide identification, 3D modeling, expression and enzymatic activity analysis of cell wall invertase gene family from cassava (*Manihot*
*esculenta* Crantz). Int J Mol Sci.

[51] Jain R, Singh SP, Singh A, Singh S, Kishor R, Singh RK (2017). Soluble acid invertase (SAI) activity and gene expression controlling sugar composition in sugarcane. Sugar Tech.

[52] Li R, Hou Z, Zou H, Wang Y, Liao X (2018). Inactivation kinetics, structural, and morphological modification of mango soluble acid invertase by high pressure processing combined with mild temperatures. Food Res Int Ott Ont.

[53] Vargas WA, Pontis HG, Salerno GL (2007). Differential expression of alkaline and neutral invertases in response to environmental stresses: characterization of an alkaline isoform as a stress-response enzyme in wheat leaves. Planta.

[54] Kim JK, Bamba T, Harada K, Fukusaki E, Kobayashi A (2007). Time-course metabolic profiling in *Arabidopsis*
*thaliana* cell cultures after salt stress treatment. J Exp Bot.

[55] Balk PA, de Boer AD (1999). Rapid stalk elongation in tulip (*Tulipa*
*gesneriana* L. cv. Apeldoorn) and the combined action of cold-induced invertase and the water-channel protein gammaTIP. Planta.

[56] Liao S, Lin C, Wang A, Sung H (2013). Differential expression of genes encoding acid invertases in multiple shoots of bamboo in response to various phytohormones and environmental factors. J Agric Food Chem.

[57] Chen C, Chen H, Zhang Y, Thomas HR, Frank MH, He Y (2020). TBtools: an integrative toolkit developed for interactive analyses of big biological data. Mol Plant.

[58] Larkin MA, Blackshields G, Brown NP, Chenna R, McGettigan PA, McWilliam H (2007). Clustal W and Clustal X version 2.0. Bioinforma Oxf Engl.

[59] Tamura K, Stecher G, Kumar S (2021). MEGA11: molecular evolutionary genetics analysis version 11. Mol Biol Evol.

[60] Kim D, Langmead B, Salzberg SL (2015). HISAT: a fast spliced aligner with low memory requirements. Nat Methods.

[61] Pertea M, Pertea GM, Antonescu CM, Chang T, Mendell JT, Salzberg SL (2015). StringTie enables improved reconstruction of a transcriptome from RNA-seq reads. Nat Biotechnol.

[62] Yuan Y, Zhang J, Liu X, Meng M, Wang J, Lin J (2020). Tissue-specific transcriptome for *Dendrobium*
*officinale* reveals genes involved in flavonoid biosynthesis. Genomics.

[63] Livak KJ, Schmittgen TD (2001). Analysis of relative gene expression data using real-time quantitative PCR and the 2^*-*^^*△△*^^*CT*^ Method. Methods San Diego Calif.

[64] Szklarczyk D, Franceschini A, Wyder S, Forslund K, Heller D, Huerta-Cepas J (2015). STRING v10: protein- protein interaction networks, integrated over the tree of life. Nucleic Acids Res.

[65] Shannon P, Markiel A, Ozier O, Baliga NS, Wang JT, Ramage D (2003). Cytoscape: a software environment for integrated models of biomolecular interaction networks. Genome Res.

