# Supplementary material for: Genomic identification and expression analysis of acid invertase (AINV) gene family in Dendrobium officinale Kimura et Migo
Source: BMC Plant Biol. 2024 May 14;24:396. doi: 10.1186/s12870-024-05102-8 (PMC11092110; doi:10.1186/s12870-024-05102-8)
Supplement: Supplementary file 1 — Supplementary Material 1. [file 12870_2024_5102_MOESM1_ESM.zip › Additional files_rev/Additioanl file 1 Figure S2.doc]

**Fig. S2.** Predicted transmembrane helix domain architectures of DoAINV proteins.
